# Oxyresveratrol Suppresses EGF-Induced AKT Phosphorylation and Reduces Cellular Fitness While Promoting Apoptosis in EGFR–Wild-Type Non-Small Cell Lung Cancer Cells Under EGF Stimulation

**DOI:** 10.3390/ijms27146403

**Published:** 2026-07-18

**Authors:** Wutigri Nimlamool, Jatuporn Polhiran, Nitchakarn Phimthong, Saranyapin Potikanond, Nitwara Wikan

**Affiliations:** 1 Department of Pharmacology, Faculty of Medicine, Chiang Mai University, Chiang Mai 50200, Thailand; wutigri.nimlamool@cmu.ac.th (W.N.); jatupornbam@gmail.com (J.P.); nitchakarn_ph@cmu.ac.th (N.P.); saranyapin.p@cmu.ac.th (S.P.); 2 Lanna Rice Research Center, Chiang Mai University, Chiang Mai 50200, Thailand; 3 PhD’s Degree Program in Pharmacology, Department of Pharmacology, Faculty of Medicine, Chiang Mai University, Chiang Mai 50200, Thailand

**Keywords:** non-small cell lung cancer, epidermal growth factor, epidermal growth factor receptor (EGFR) signaling, phosphoinositide 3-kinase/protein kinase B (PI3K/AKT) pathway, oxyresveratrol, apoptosis, natural compounds

## Abstract

Lung cancer remains the leading cause of cancer-related mortality worldwide, with non-small cell lung cancer (NSCLC) accounting for the majority of cases and frequently associated with poor outcomes due to resistance to conventional therapies. The epidermal growth factor (EGF)–epidermal growth factor receptor (EGFR) axis plays a central role in NSCLC progression by activating downstream phosphoinositide 3-kinase/protein kinase B (PI3K/AKT) signaling, thereby promoting survival and proliferation. Natural compounds have emerged as promising modulators of these pathways, and oxyresveratrol (OXY), a hydroxylated analog of resveratrol, has been reported to possess antioxidant and anticancer properties, though its mechanistic role in NSCLC remains unclear. In this study, we investigated the effects of OXY in EGF-stimulated A549 and H1299 cells. OXY significantly reduced metabolic activity and decreased cell number in a dose-dependent manner and increased the proportion of apoptotic cells. Mechanistically, OXY selectively attenuated EGF-induced AKT phosphorylation while largely sparing extracellular signal–regulated kinase 1/2 (ERK1/2) activation and did not measurably alter EGFR phosphorylation or receptor trafficking dynamics. These findings indicate that OXY exposure is associated with AKT-selective signaling suppression and reduced cellular fitness in NSCLC cells, without evidence of direct EGFR inhibition. Further genetic rescue and pathway-epistasis studies are required to establish causal dependency on AKT signaling and to support in vivo validation.

## 1. Introduction

Lung cancer remains the leading cause of cancer-related mortality worldwide [1]. This persistent burden underscores the urgent need for improved therapeutic strategies that address both tumor biology and treatment resistance. Among histological subtypes, non-small cell lung cancer (NSCLC) represents the majority of cases and is frequently associated with poor clinical outcomes, largely due to late-stage diagnosis, limited treatment options, and the emergence of resistance to conventional therapies [2].

The epidermal growth factor (EGF)–epidermal growth factor receptor (EGFR) axis is a central regulator of tumor cell biology, orchestrating survival and proliferation [3]. Activation of EGFR signaling fosters oncogenesis through downstream cascades such as the phosphoinositide 3-kinase (PI3K)/protein kinase B (AKT) and mitogen-activated protein kinase (MAPK) pathways [4]. Notably, EGF stimulation induces AKT activation in NSCLC cells, reinforcing survival signaling. The PI3K/AKT pathway has thus emerged as a critical mediator of cancer cell survival, and its inhibition has been shown to enhance chemotherapy-induced apoptosis [5,6]. Given the molecular heterogeneity of NSCLC, evaluating candidate agents that modulate PI3K/AKT signaling in defined cellular experimental models remains important for clarifying their biological effects and potential scope of activity.

Natural compounds have gained increasing attention as potential anticancer agents due to their ability to modulate multiple signaling pathways with relatively low toxicity. Several plant-derived compounds, including flavonoids such as luteolin [7] and apigenin [8], as well as alkaloids like berberine [9] and terpenoids such as tanshinone IIA [10], have been shown to inhibit PI3K/AKT signaling in NSCLC, suppressing proliferation, inducing apoptosis, and sensitizing tumor cells to chemotherapy.

Among stilbenes, resveratrol has been shown to inhibit PI3K/AKT signaling in NSCLC, reducing proliferation and enhancing apoptosis [11]. Oxyresveratrol (OXY), a structurally related stilbene predominantly found in mulberries and other plant sources [12,13], exhibits pharmacological properties. Its diverse biological activities include anti-inflammatory, neuroprotective, and anticancer effects [14,15,16,17,18,19]. More recently, evidence indicates that OXY can negatively regulate PI3K/AKT signaling, thereby impairing tumor cell survival and proliferation [20,21]. Despite these promising findings, OXY remains underexplored in the context of EGF-induced signaling in NSCLC.

The present study was designed to investigate the pharmacological activity of OXY in NSCLC cells. Specifically, we examined the effects of OXY on EGF-stimulated PI3K/AKT pathway activity and associated cellular outcomes. Using EGFR–wild-type lung cancer cell models (A549 and H1299), we assessed whether OXY treatment is accompanied by reduced AKT phosphorylation and concurrent decreased metabolic activity and decreased cell number, as well as pro-apoptotic effects. Together, these experiments aim to characterize the relationship between PI3K/AKT pathway modulation and OXY-associated cellular responses in NSCLC, providing a foundation for future mechanistic studies and validation in additional disease-relevant models.

## 2. Results

### 2.1. Oxyresveratrol (OXY) Attenuates Metabolic Activity in Non-Small Cell Lung Cancer (NSCLC) Cells

Assessing metabolic activity is a critical step in evaluating the potential anticancer properties of candidate compounds, as it provides direct insight into cell metabolic state/fitness under treatment conditions. The 3-(4,5-dimethylthiazol-2-yl)-2,5-diphenyltetrazolium bromide (MTT) assay, widely regarded as a reliable measure of mitochondrial function and metabolic status [22], was therefore employed to determine the impact of OXY on NSCLC cells. This approach allowed us to quantify dose-dependent changes in cellular activity and establish whether OXY could effectively suppress NSCLC cell metabolic activity (MTT signal) in the presence of growth stimuli.

**Figure 1:**
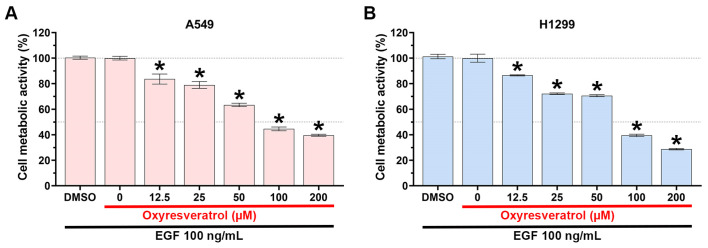
Effect of oxyresveratrol (OXY) on epidermal growth factor (EGF)-stimulated metabolic activity of non-small cell lung cancer (NSCLC) cell lines. 3-(4,5-dimethylthiazol-2-yl)-2,5-diphenyltetrazolium bromide (MTT) assay showing that OXY reduced metabolic activity of A549 (**A**) and H1299 (**B**) cells after 48 h. Groups on the x-axis are dimethyl sulfoxide (DMSO) vehicle and OXY (0, 12.5, 25, 50, 100, 200 µM) under EGF stimulation. Data are presented as mean ± standard error of the mean (SEM) of *n* = 3 independent biological replicates per group (*N* = 21 values per cell line). Statistical analysis was performed using ordinary one-way analysis of variance (ANOVA) followed by Dunnett’s multiple comparisons test comparing each condition to 0 µM OXY (EGF alone). For A549, ANOVA showed a significant treatment effect (F(6,14) = 137.7, *p* < 0.0001); Dunnett-adjusted *p*-values vs. 0 µM OXY were: 12.5 µM (*p* = 0.0004), 25 µM (*p* < 0.0001), 50 µM (*p* < 0.0001), 100 µM (*p* < 0.0001), and 200 µM (*p* < 0.0001), whereas DMSO was not significant (*p* = 0.9998). For H1299, ANOVA showed a significant treatment effect (F(6,14) = 356.5, *p* < 0.0001); Dunnett-adjusted *p*-values vs. 0 µM OXY were: 12.5 µM (*p* = 0.0001), 25 µM (*p* < 0.0001), 50 µM (*p* < 0.0001), 100 µM (*p* < 0.0001), and 200 µM (*p* < 0.0001), whereas DMSO was not significant (*p* = 0.9717). * *p* < 0.05 vs. 0 µM OXY (Dunnett-adjusted); comparisons without asterisks are not significant (ns) (Dunnett-adjusted).

OXY significantly inhibited metabolic activity in NSCLC cell lines cultured in the presence of epidermal growth factor (EGF). MTT assays demonstrated a clear, dose-dependent reduction in metabolic activity in both A549 and H1299 cells after 48 h of treatment with OXY, whereas no effect was observed with the dimethyl sulfoxide (DMSO) vehicle control (Figure 1A,B). Compared with untreated controls, OXY exposure led to a progressive decline in cell metabolic activity, and statistical analysis confirmed significant reductions at all concentrations. Dose–response curve fitting at 48 h yielded MTT-derived half-maximal inhibitory concentration (IC50) values of 98.95 μM for A549 cells and 80.54 μM for H1299 cells (Figure 1A,B).

**Figure 2:**
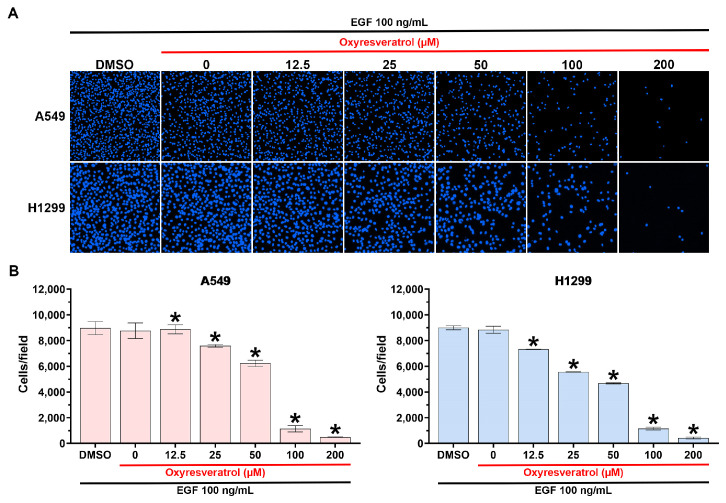
Effect of OXY on NSCLC cell number. (**A**) Representative Hoechst 33342-stained images (magnification: 4×) of nuclei (blue) from A549 and H1299 cells treated with the indicated concentrations of OXY under EGF stimulation for 48 h. (**B**) Quantification of Hoechst-positive nuclei showing that OXY reduced cell number in both A549 and H1299 cells in a dose-dependent manner after 48 h. Data are presented as mean ± SEM of *n* = 3 independent biological replicates per group (*N* = 21 values per cell line). Statistical analysis was performed using ordinary one-way ANOVA followed by Dunnett’s multiple comparisons test (each condition compared with 0 µM OXY [EGF alone]; family-wise alpha (α) = 0.05; multiplicity-adjusted). For A549, one-way ANOVA indicated a significant treatment effect (F(6,14) = 106.8, *p* < 0.0001); Dunnett-adjusted comparisons vs. 0 µM OXY were significant for 50 µM (*p* = 0.0009), 100 µM (*p* < 0.0001), and 200 µM (*p* < 0.0001). For H1299, one-way ANOVA indicated a significant treatment effect (F(6,14) = 699.8, *p* < 0.0001); Dunnett-adjusted comparisons vs. 0 µM OXY were significant for 12.5–200 µM (all *p* < 0.0001). * *p* < 0.05 vs. 0 µM OXY (Dunnett-adjusted); comparisons without asterisks are ns (Dunnett-adjusted).

Consistently, OXY treatment significantly reduced the number of NSCLC cells in a dose-dependent manner. Hoechst 33342 staining revealed a marked decrease in the number of nuclei in both A549 and H1299 cells after 48 h of exposure to increasing concentrations of OXY in the presence of EGF (Figure 2A). Quantitative cell counts corroborated these findings, showing that OXY—but not the DMSO vehicle control—led to a progressive reduction in cell number compared with untreated controls (Figure 2B). Dose–response curve fitting at 48 h yielded cell count-derived IC50 values of 61.99 μM for A549 cells and 40.25 μM for H1299 cells (Figure 2B).

### 2.2. OXY Induces Apoptosis in NSCLC Cells

**Figure 3:**
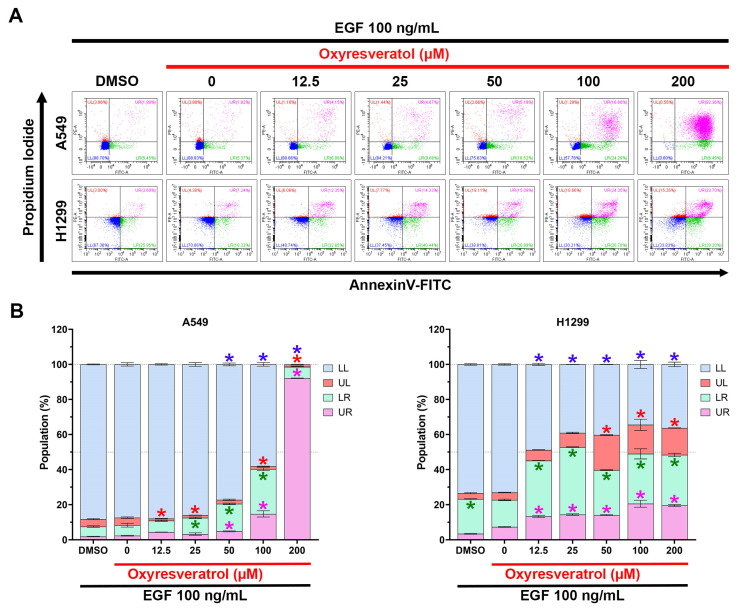
Effect of OXY on apoptosis in EGF-stimulated NSCLC cells. Annexin V– fluorescein isothiocyanate (FITC)/propidium iodide (PI) staining followed by flow cytometry was performed in A549 and H1299 cells treated with the indicated concentrations of OXY under EGF stimulation. (**A**) Representative dot plots showing four populations defined by quadrant gating: lower left (LL), viable (Annexin V−/PI−); lower right (LR), early apoptotic (Annexin V+/PI−); upper right (UR), late apoptotic/secondary necrotic (Annexin V+/PI+); upper left (UL), primary necrotic (Annexin V−/PI+). Colored asterisks within the dot plots indicate quadrant identity (blue, LL; red, UL; green, LR; pink, UR). (**B**) Quantification of the percentage of cells in each quadrant; total apoptotic cells were calculated as LR + UR. Data are presented as mean ± SEM from *n* = 3 independent biological replicates per condition. Statistical analysis was performed using an ordinary two-way ANOVA (treatment × quadrant) followed by Dunnett’s multiple comparisons test (within each quadrant, each condition compared with 0 µM OXY [EGF alone]; family-wise α = 0.05; multiplicity-adjusted). For A549, a significant treatment × quadrant interaction was detected (F(18,56) = 1851, *p* < 0.0001); the quadrant main effect was significant (F(3,56) = 16,269, *p* < 0.0001), whereas the treatment main effect was not significant (F(6,56) = 3.363 × 10^−6^, *p* > 0.9999). Significant vs. control (Dunnett-adjusted *p*-values): UR—50 (*p* = 0.0398), 100 (*p* < 0.0001), 200 (*p* < 0.0001); UL—12.5 (*p* = 0.0055), 25 (*p* = 0.0128), 100 (*p* = 0.0166), 200 (*p* = 0.0007); LL—50 (*p* < 0.0001), 100 (*p* < 0.0001), 200 (*p* < 0.0001); LR—25 (*p* = 0.0025), 50 (*p* < 0.0001), 100 (*p* < 0.0001). For H1299, a significant treatment × quadrant interaction was detected (F(18,56) = 119.5, *p* < 0.0001); the quadrant main effect was significant (F(3,56) = 1853, *p* < 0.0001), whereas the treatment main effect was not significant (F(6,56) = 2.332 × 10^−6^, *p* > 0.9999). Significant vs. control (Dunnett-adjusted *p*-values): UR—12.5 (*p* = 0.0014), 25 (*p* = 0.0002), 50 (*p* = 0.0003), 100 (*p* < 0.0001), 200 (*p* < 0.0001); UL—50 (*p* < 0.0001), 100 (*p* < 0.0001), 200 (*p* < 0.0001); LL—12.5–200 (all *p* < 0.0001); LR—DMSO (*p* = 0.0288), 12.5–200 (all *p* < 0.0001). Asterisks above bars indicate statistical significance (* *p* < 0.05 vs. 0 µM OXY [EGF alone] within the same quadrant; Dunnett-adjusted); comparisons without asterisks are ns (Dunnett-adjusted). (**Left**): A549; (**Right**): H1299.

The reduction in cell metabolic activity and cell number observed in A549 and H1299 cells following OXY treatment suggested that apoptosis might be involved. To test this hypothesis, we performed Annexin V–fluorescein isothiocyanate (FITC)/propidium iodide (PI) staining followed by flow cytometry to evaluate apoptosis in the presence of EGF and to distinguish apoptotic from necrotic populations. As shown in Figure 3, OXY treatment significantly increased the proportion of Annexin V-positive cells compared with EGF-stimulated controls, reflected by increases in early apoptotic (Annexin V+/PI−) and late apoptotic/secondary necrotic (Annexin V+/PI+) populations even under conditions that normally promote cell survival. This pro-apoptotic effect was consistently observed in both A549 and H1299 cells, supporting the conclusion that OXY counteracts EGF-mediated survival signaling in NSCLC cells. To distinguish apoptosis from necrosis, Annexin V–FITC/PI data were analyzed by quadrant gating to quantify viable, early apoptotic, late apoptotic/secondary necrotic, and primary necrotic populations [viable (LL), Annexin V−/PI−; early apoptotic (LR), Annexin V+/PI−; late apoptotic/secondary necrotic (UR), Annexin V+/PI+; primary necrotic (UL), Annexin V−/PI+] (Figure 3A,B), and total apoptotic cells were calculated as LR + UR.

**Figure 4:**
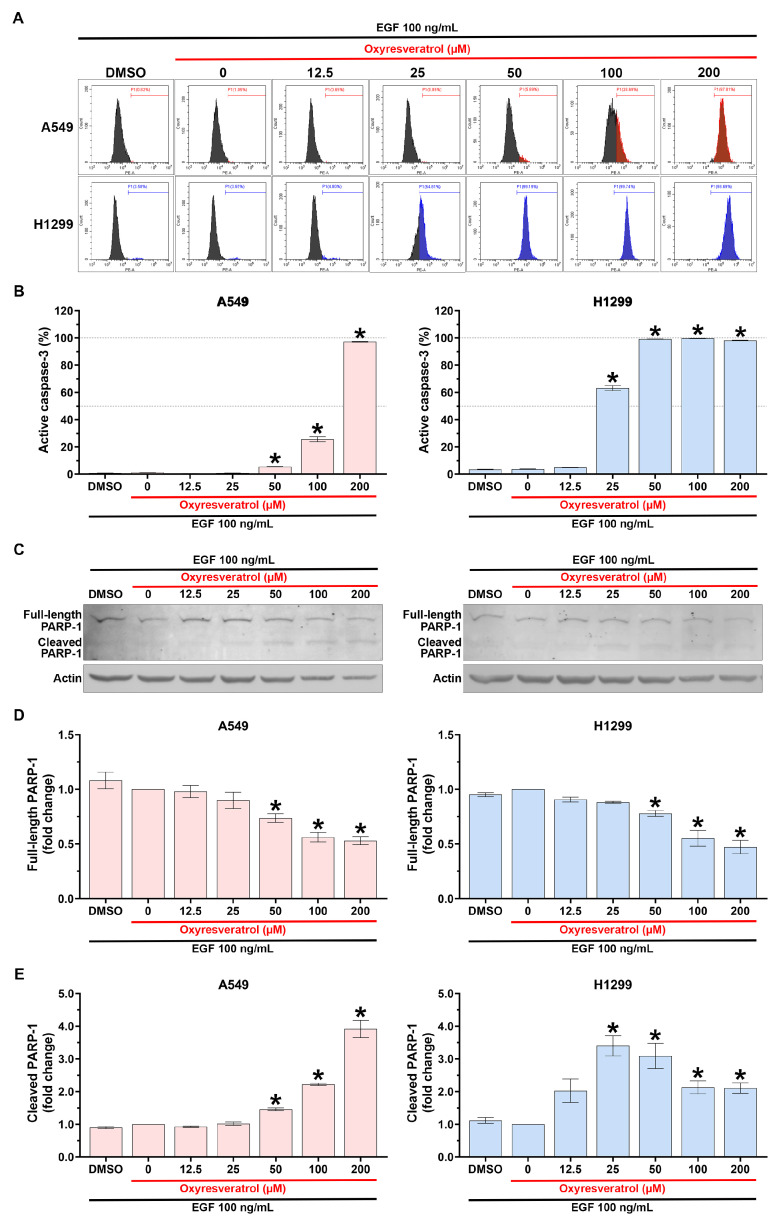
OXY induces apoptosis in A549 and H1299 cells, as evidenced by increased active caspase-3 and poly(ADP-ribose) polymerase 1 (PARP-1) cleavage. (**A**) Representative flow-cytometry histograms of phycoerythrin (PE)-active caspase-3 staining in A549 and H1299 cells treated with the indicated concentrations of OXY under EGF stimulation (PE Active Caspase-3 Apoptosis Kit); a rightward shift indicates an increased active caspase-3 signal. (**B**) Quantification of active caspase-3-positive cells, expressed as % of 0 µM OXY (EGF alone) (set to 100%). Data are presented as mean ± SEM from *n* = 6 independent biological replicates per group (*N* = 42 per cell line). Statistical analysis was performed using ordinary one-way ANOVA followed by Dunnett’s multiple comparisons test (each condition vs. 0 µM OXY [EGF alone]; family-wise α = 0.05; multiplicity-adjusted). For A549, ANOVA showed a significant treatment effect (F(6,35) = 2548, *p* < 0.0001); significant vs. control: 50 µM (adjusted *p* = 0.0009), 100 µM (adjusted *p* < 0.0001), and 200 µM (adjusted *p* < 0.0001). For H1299, ANOVA showed a significant treatment effect (F(6,34) = 4386, *p* < 0.0001); significant vs. control: 25–200 µM (all adjusted *p* < 0.0001). (**C**) Representative Western blots of PARP-1 (full-length and cleaved) in A549 and H1299 cells following OXY treatment ((**Left**): A549; (**Right**): H1299). (**D**,**E**) Densitometric quantification of full-length PARP-1 (**D**) and cleaved PARP-1 (**E**) after OXY treatment. Cells were treated with 0 µM OXY (EGF alone; control set to 100%), OXY (12.5, 25, 50, 100, or 200 µM), and a DMSO vehicle control. Band intensities were normalized to beta (β)-actin and expressed as % of 0 µM OXY (EGF alone). Data are presented as mean ± SEM from *n* = 3 independent biological replicates per group. Statistical analysis was performed using ordinary one-way ANOVA followed by Dunnett’s multiple comparisons test (each condition vs. 0 µM OXY [EGF alone]; family-wise α = 0.05; multiplicity-adjusted). For full-length PARP-1 (**D**), significant vs. control: A549—50 (*p* = 0.0154), 100 (*p* = 0.0002), 200 (*p* = 0.0001); H1299—50 (*p* = 0.0063), 100 (*p* < 0.0001), 200 (*p* < 0.0001). For cleaved PARP-1 (E), significant vs. control: A549—50 (*p* = 0.0369), 100 (*p* < 0.0001), 200 (*p* < 0.0001); H1299—25 (*p* < 0.0001), 50 (*p* = 0.0002), 100 (*p* = 0.0311), 200 (*p* = 0.0346). * *p* < 0.05 vs. 0 µM OXY within the same panel (Dunnett-adjusted); comparisons without asterisks are ns.

To further confirm that OXY induces apoptosis, active (cleaved) caspase-3 was measured by flow cytometry using the phycoerythrin (PE) Active Caspase-3 Apoptosis Kit following treatment with increasing concentrations of OXY. As shown in Figure 4A, OXY treatment caused a clear rightward shift in the PE fluorescence peak in both A549 and H1299 cells, consistent with increased active caspase-3 levels. The corresponding quantification is presented in Figure 4B as the percentage of active caspase-3-positive cells, expressed as percent of the EGF-treated control (OXY 0 µM; set to 100%). In A549 cells, the active caspase-3-positive fraction was significantly increased at 50, 100, and 200 µM OXY. In H1299 cells, active caspase-3 positivity was significantly increased at 25, 50, 100, and 200 µM, indicating activation at lower OXY concentrations compared with A549. To further corroborate caspase-dependent apoptosis, we next examined poly(ADP-ribose) polymerase 1 (PARP-1) cleavage by Western blotting following OXY treatment. Consistent with the active caspase-3 flow-cytometry results, OXY exposure led to a marked apoptotic PARP-1 processing pattern in both cell lines. Specifically, the full-length PARP-1 band was significantly reduced at 50, 100, and 200 µM OXY in both A549 and H1299 cells (Figure 4C,D). Concomitantly, the cleaved PARP-1 fragment was significantly increased in a concentration-dependent manner (Figure 4C,E). In A549 cells, cleaved PARP-1 was significantly elevated at 50, 100, and 200 µM, with the strongest increase observed at 200 µM (Figure 4E). In H1299 cells, cleaved PARP-1 increased significantly at 25, 50, 100, and 200 µM, reaching a maximum at 25 µM (Figure 4E). Together with the increased active caspase-3 signal, these data provide additional biochemical evidence that OXY induces apoptotic cell death in both A549 and H1299 cells.

### 2.3. Lack of Inhibitory Effect of OXY on Epidermal Growth Factor Receptor (EGFR) Activation in NSCLC Cells Following EGF Stimulation

**Figure 5:**
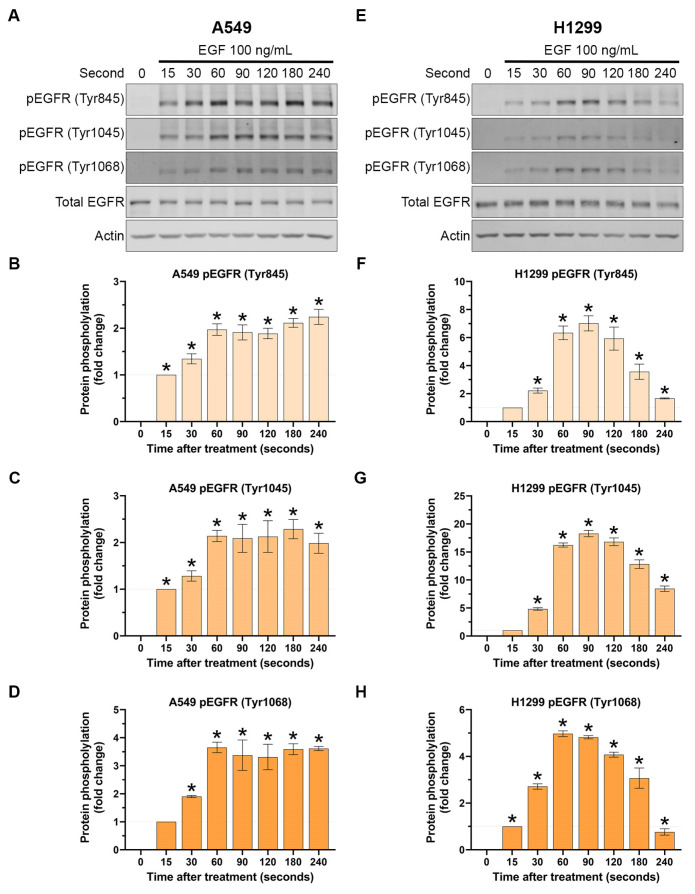
Temporal dynamics of EGFR activation in NSCLC cells following EGF stimulation. (**A**) Representative immunoblots showing EGFR phosphorylation at Tyr845, Tyr1045, and Tyr1068, together with total EGFR and actin, in A549 cells at the indicated times after EGF stimulation. (**B**–**D**) Densitometric quantification of phosphorylated EGFR (pEGFR) Tyr845 (**B**), pEGFR Tyr1045 (**C**), and pEGFR Tyr1068 (**D**) in A549 cells, normalized to total EGFR and expressed relative to 0 minutes (min). (**E**) Representative immunoblots showing EGFR phosphorylation at Tyr845, Tyr1045, and Tyr1068, together with total EGFR and actin, in H1299 cells at the indicated times after EGF stimulation. (**F**–**H**) Densitometric quantification of pEGFR Tyr845 (**F**), pEGFR Tyr1045 (**G**), and pEGFR Tyr1068 (**H**) in H1299 cells, normalized to total EGFR and expressed relative to 0 min. Data are presented as mean ± SEM from *n* = 3 independent experiments. For each phosphorylation-site time course, statistical analysis was performed using ordinary one-way ANOVA followed by Dunnett’s multiple comparisons test (each time point vs. 0 min, multiplicity-adjusted). All ANOVAs: *p* < 0.0001. ns vs. 0 min (Dunnett-adjusted): A549 pTyr1068—15 min (*p* = 0.0872); H1299 pTyr845—15 min (*p* = 0.4551) and 240 min (*p* = 0.0791); H1299 pTyr1045—15 min (*p* = 0.5408). All other comparisons vs. 0 min were significant (adjusted *p* < 0.05). * *p* < 0.05 vs. 0 min (Dunnett-adjusted); comparisons without asterisks are ns.

We characterized the temporal dynamics of EGFR activation following EGF stimulation by assessing receptor phosphorylation at multiple tyrosine (Tyr) residues (Tyr845, Tyr1045, and Tyr1068). As shown in Figure 5, EGF treatment induced rapid phosphorylation at these sites, with detectable phosphorylation evident as early as 15 seconds (s) post-stimulation. In A549 cells, phosphorylation at all three residues was sustained for up to 240 s (Figure 5A–D), whereas in H1299 cells phosphorylation declined after 90 s and reached minimal levels by 240 s (Figure 5E–H). These findings confirm that both A549 and H1299 cells retain functional wild-type (WT) EGFR signaling capacity upon EGF stimulation. We therefore selected the 60-s peak of EGF stimulation as the experimental time point to evaluate the effect of OXY on phosphorylation of these EGFR tyrosine residues.

**Figure 6:**
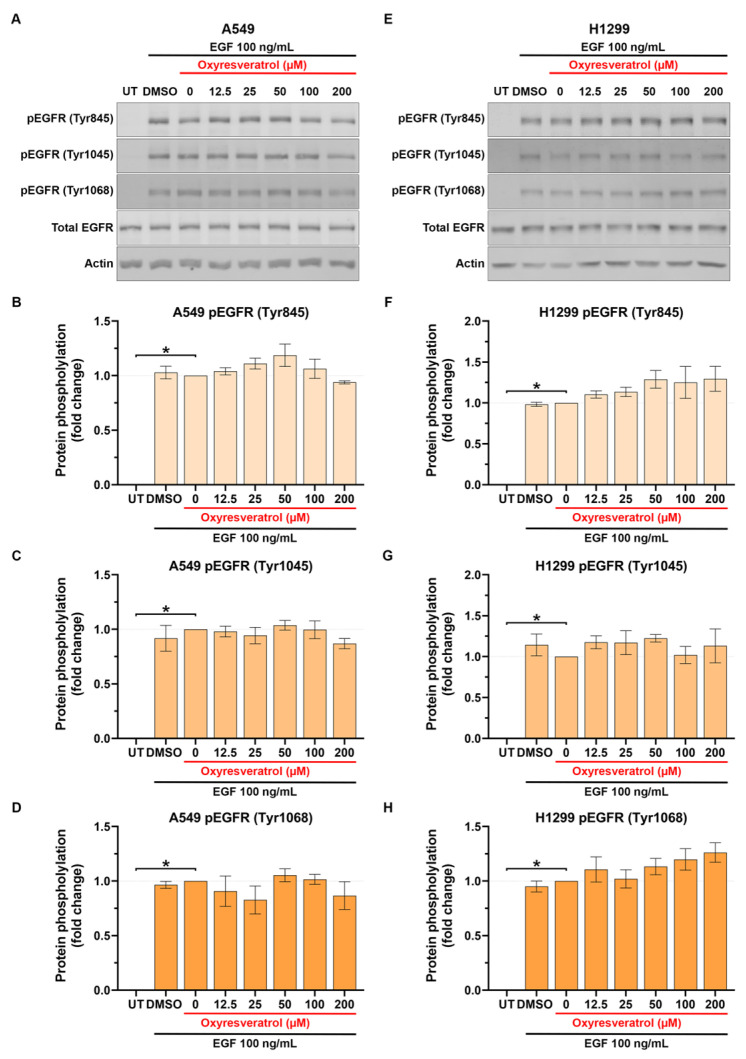
Effect of OXY on EGFR phosphorylation in NSCLC cells. (**A**) Representative immunoblots of pEGFR (Tyr845, Tyr1045, Tyr1068), total EGFR, and actin in A549 cells following 3 h OXY pretreatment and stimulation with 100 ng/mL EGF for 60 s. (**B**–**D**) Densitometric quantification of pEGFR Tyr845 (**B**), pEGFR Tyr1045 (**C**), and pEGFR Tyr1068 (**D**) in A549 cells, normalized to total EGFR and expressed relative to 0 µM OXY (EGF alone). (**E**) Representative immunoblots in H1299 cells under the same conditions. (**F**–**H**) Densitometric quantification of pEGFR Tyr845 (**F**), pEGFR Tyr1045 (**G**), and pEGFR Tyr1068 (**H**) in H1299 cells, normalized to total EGFR and expressed relative to 0 µM OXY (EGF alone). Data are presented as mean ± SEM from *n* = 3 independent biological replicates per group. For each panel (**B**–**D**,**F**–**H**), statistical analysis was performed using ordinary one-way ANOVA followed by Dunnett’s multiple comparisons test (each condition vs. 0 µM OXY [EGF alone]; family-wise α = 0.05; multiplicity-adjusted). One-way ANOVA was significant for all panels: **B** F(7,16) = 45.06, *p* < 0.0001 (R^2^ = 0.9517); **C** F(7,16) = 28.99, *p* < 0.0001 (R^2^ = 0.9269); **D** F(7,16) = 16.15, *p* < 0.0001 (R^2^ = 0.8760); **F** F(7,16) = 18.33, *p* < 0.0001 (R^2^ = 0.8891); **G** F(7,16) = 12.91, *p* < 0.0001 (R^2^ = 0.8496); **H** F(7,16) = 27.94, *p* < 0.0001 (R^2^ = 0.9244). Dunnett-adjusted comparisons vs. 0 µM OXY (EGF alone) indicated untreated (UT) was significant in all panels (adjusted *p* < 0.0001), whereas DMSO and OXY (12.5–200 µM) were not significant in any panel (all adjusted *p* ≥ 0.1234). * *p* < 0.05 vs. 0 µM OXY (EGF alone) (Dunnett-adjusted); comparisons without asterisks are ns.

Untreated A549 and H1299 cells exhibited no detectable phosphorylation of EGFR at Tyr845, Tyr1045, or Tyr1068, although total EGFR levels remained consistent across all conditions (Figure 6A–H). Stimulation with 100 ng/mL EGF induced robust phosphorylation at all three tyrosine residues in both cell lines (Figure 6A–H). Notably, pretreatment with OXY failed to suppress EGF-induced phosphorylation at Tyr845, Tyr1045, or Tyr1068, and did not alter total EGFR levels (Figure 6A–H), suggesting that OXY does not interfere with EGF-dependent EGFR activation.

**Figure 7:**
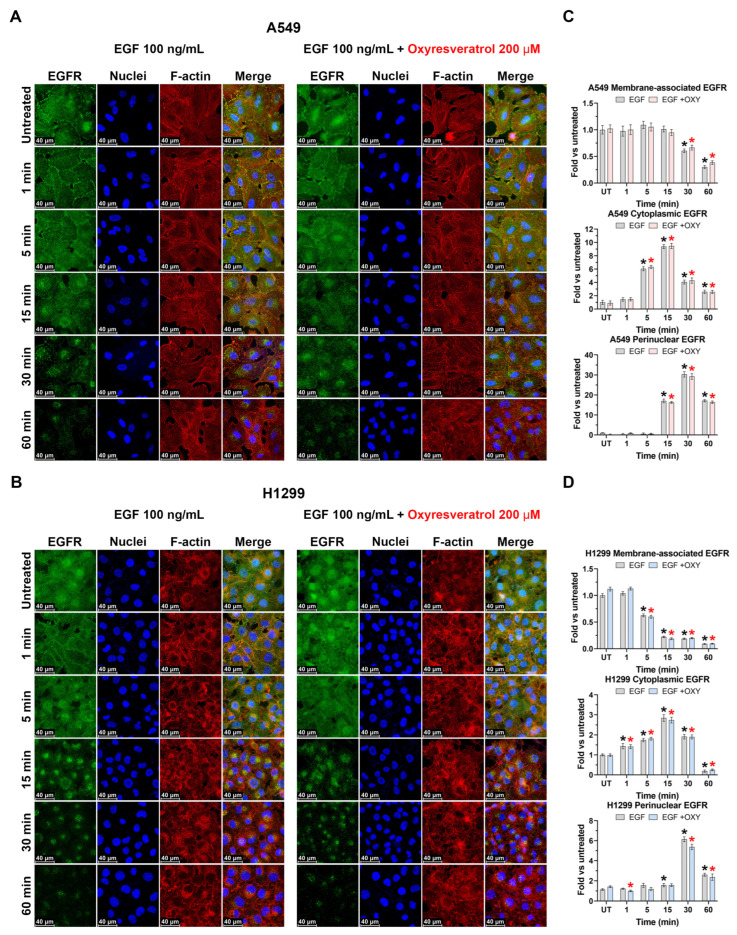
Effect of OXY on EGF-induced EGFR trafficking in NSCLC cells. (**A**) Representative immunofluorescence images of A549 cells stimulated with EGF (100 ng/mL) for the indicated times (1–60 min), showing EGFR (green) internalization toward the perinuclear region and a progressive reduction in signal over time, with or without OXY pretreatment (200 µM, 3 h). (**B**) Representative images of H1299 cells under the same conditions. Nuclei were counterstained with 4′,6-diamidino-2-phenylindole (DAPI) (blue), and F-actin was labeled with phalloidin (red). Scale bar, 40 µm (100×). (**C**,**D**) Quantification of EGFR localization in A549 (**C**) and H1299 (**D**) cells. Background-corrected EGFR fluorescence intensity was quantified per cell in membrane-associated, cytoplasmic, and perinuclear compartments at each time point and summarized across *n* = 3 independent biological replicates. Data are presented as mean ± SEM. Statistics (**C**,**D**): Data were analyzed using a mixed-effects model (REML) with Geisser–Greenhouse correction, with time and treatment (EGF vs. EGF + OXY) as fixed effects and subject as a random effect (family-wise α = 0.05; multiplicity-adjusted). For A549 (**C**), time significantly affected membrane-associated EGFR (F(3.344,131.1) = 39.66, *p* < 0.0001), cytoplasmic EGFR (F(4.225,264.5) = 213.9, *p* < 0.0001), and perinuclear EGFR (F(2.261,175.5) = 641.9, *p* < 0.0001), whereas treatment and time × treatment effects were not significant (membrane: *p* = 0.8726 and *p* = 0.8335; cytoplasmic: *p* = 0.6516 and *p* = 0.9912; perinuclear: *p* = 0.2145 and *p* = 0.8328). For H1299 (**D**), time significantly affected membrane-associated EGFR (F(3.112,189.2) = 600.6, *p* < 0.0001), cytoplasmic EGFR (F(3.189,191.3) = 153.4, *p* < 0.0001), and perinuclear EGFR (F(3.049,231.8) = 214.7, *p* < 0.0001). The treatment main effect was significant for perinuclear EGFR (F(1,125) = 4.512, *p* = 0.0356) but not for membrane-associated or cytoplasmic EGFR (*p* = 0.1453 and *p* = 0.8965). The time × treatment interaction was significant for membrane-associated EGFR (F(5,304) = 3.013, *p* = 0.0114) but not for cytoplasmic (*p* = 0.9572) or perinuclear EGFR (*p* = 0.0543). Šídák-adjusted comparisons between EGF and EGF + OXY at individual time points were not significant in any compartment (all adjusted *p* ≥ 0.1168). Significance notation (**C**,**D**): Black asterisks indicate *p* < 0.05 vs. UT within the EGF group; red asterisks indicate *p* < 0.05 vs. UT within the EGF + OXY group (multiplicity-adjusted; family-wise α = 0.05). Unmarked comparisons are not significant.

To further examine the effect of OXY on the temporal kinetics of EGFR trafficking, we performed immunofluorescence analysis following EGF stimulation (Figure 7). As shown in Figure 7A,B, unstimulated A549 and H1299 cells displayed a diffuse EGFR distribution throughout the cell, with prominent staining at cell–cell contact regions. Upon stimulation with 100 ng/mL EGF, both cell lines showed time-dependent EGFR internalization toward the perinuclear region, followed by a progressive reduction in EGFR signal over 60 min, consistent with receptor trafficking and degradation (Figure 7A,B). Pretreatment with 200 µM OXY for 3 h produced a broadly comparable pattern of EGFR endocytosis, relocalization, and signal reduction in both cell lines (Figure 7A,B). Quantitative compartment-based analysis (membrane-associated, cytoplasmic, and perinuclear EGFR) confirmed a strong effect of time in A549 and H1299 (all compartments, *p* < 0.0001; Figure 7C,D). In A549 cells, neither the treatment main effect nor the time × treatment interaction was significant for any compartment, indicating no detectable effect of OXY on EGFR trafficking kinetics (Figure 7C). In H1299 cells, although a modest treatment main effect was observed for perinuclear EGFR and a time × treatment interaction was detected for membrane-associated EGFR, Šídák-adjusted comparisons between EGF and EGF + OXY at individual time points were not significant (all adjusted *p* ≥ 0.1168), supporting the conclusion that OXY does not robustly alter EGF-driven EGFR trafficking under these conditions (Figure 7D).

### 2.4. OXY Specifically Suppresses EGF-Stimulated AKT Phosphorylation in NSCLC Cells

**Figure 8:**
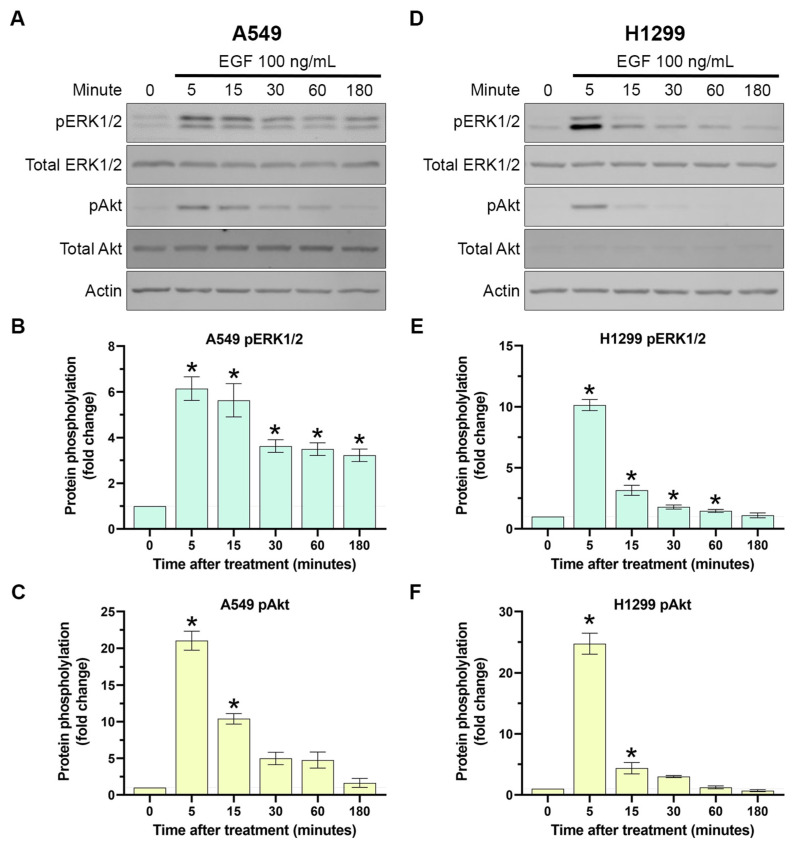
EGF-induced activation kinetics of ERK1/2 and AKT signaling in NSCLC cells. (**A**) Representative immunoblots of phosphorylated ERK1/2 (pERK1/2), total ERK1/2, phosphorylated AKT (pAKT), and total AKT in A549 cells at the indicated times after EGF stimulation. (**B**,**C**) Densitometric quantification of pERK1/2 (**B**) and pAKT (**C**) in A549 cells, normalized to the corresponding total protein and expressed relative to 0 min. (**D**) Representative immunoblots of pERK1/2, total ERK1/2, pAKT, and total AKT in H1299 cells under the same conditions. (**E**,**F**) Densitometric quantification of pERK1/2 (**E**) and pAKT (**F**) in H1299 cells, normalized to the corresponding total protein and expressed relative to 0 min. Data are presented as mean ± SEM from *n* = 3 independent biological replicates per time point. Statistics (**B**,**C**,**E**,**F**): Densitometric values were normalized to the corresponding total protein and expressed relative to 0 min. Statistical analysis was performed using ordinary one-way ANOVA followed by Dunnett’s multiple comparisons test (each time point vs. 0 min; family-wise α = 0.05; multiplicity-adjusted). One-way ANOVA showed a significant effect of time in all panels: A549 pERK1/2 (**B**) F(5,12) = 19.99, *p* < 0.0001; A549 pAKT (**C**) F(5,12) = 75.20, *p* < 0.0001; H1299 pERK1/2 (**E**) F(5,12) = 162.6, *p* < 0.0001; H1299 pAKT (**F**) F(5,12) = 133.5, *p* < 0.0001. Dunnett-adjusted comparisons vs. 0 min indicated significant increases at 5 and 15 min in all panels (all adjusted *p* ≤ 0.0457). Additional significant increases were observed in A549 at 30 min ((**B**): adjusted *p* = 0.0031; (**C**): adjusted *p* = 0.0266) and 60 min ((**B**): adjusted *p* = 0.0045; (**C**): adjusted *p* = 0.0368), and at 180 min for A549 pERK1/2 ((**B**): adjusted *p* = 0.0103); other time points were not significant. Significance notation: * *p* < 0.05 vs. 0 min (Dunnett-adjusted); unmarked comparisons are not significant.

The observation that OXY did not inhibit EGF-induced EGFR phosphorylation or alter total EGFR expression prompted further investigation into whether OXY modulates EGFR signaling indirectly through downstream effectors. We therefore examined its impact on downstream signaling cascades, specifically the mitogen-activated protein kinase (MAPK) and phosphoinositide 3-kinase (PI3K)/protein kinase B (AKT) pathways, which are central regulators of cancer cell proliferation and survival. To define the kinetics of maximal extracellular signal-regulated kinase 1/2 (ERK1/2) and AKT activation following EGF stimulation, we conducted a time-course study in A549 and H1299 cells, monitoring phosphorylation levels at multiple intervals after ligand addition. As shown in Figure 8A–F, phosphorylation of ERK1/2 and AKT in A549 and H1299 cells was rapidly induced following EGF stimulation, with both pathways reaching maximal activation at approximately 5 min post-exposure. After this peak, phosphorylation levels progressively declined over the course of 180 min. Based on the temporal dynamics of EGF-induced ERK1/2 and AKT phosphorylation, we selected 5 min post-EGF stimulation as the optimal time point for assessing the modulatory activity of OXY on the phosphorylation status of these two kinases. This time point corresponded to maximal activation of both pathways, thereby providing a robust experimental window to evaluate the capacity of OXY to interfere with downstream signaling events.

**Figure 9:**
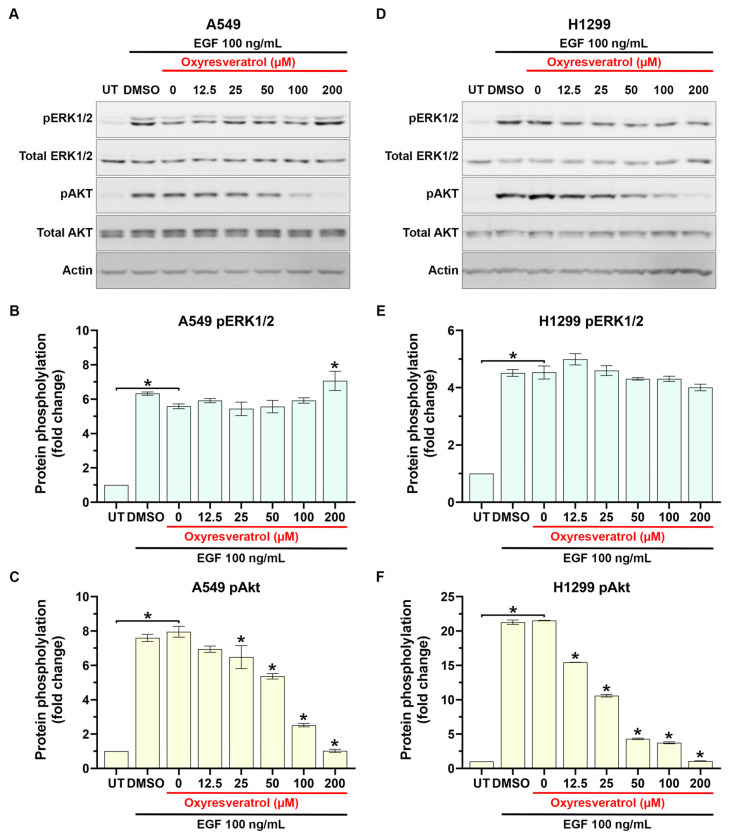
Effect of OXY on EGF-induced ERK1/2 and AKT phosphorylation in NSCLC cells. A549 and H1299 cells were pretreated with OXY for 3 h and stimulated with 100 ng/mL EGF for 5 min. Representative immunoblots are shown for A549 (**A**) and H1299 (**D**). Densitometric quantification of pERK1/2 (A549: (**B**); H1299: (**E**)) and pAKT (A549: (**C**); H1299: (**F**)) was normalized to the corresponding total protein and expressed relative to the EGF-only control (0 µM OXY + EGF). Data are presented as mean ± SEM from *n* = 3 independent biological replicates per condition. Statistics (**B**,**C**,**E**,**F**): Statistical analysis was performed using ordinary one-way ANOVA followed by Dunnett’s multiple comparisons test (each condition vs. EGF-only control; family-wise α = 0.05; multiplicity-adjusted). The UT vs. EGF-only comparison is indicated by connecting lines in the graphs. One-way ANOVA indicated significant treatment effects in all panels: A549 pERK1/2 (**B**) F(7,16) = 40.39, *p* < 0.0001; A549 pAKT (**C**) F(7,16) = 101.1, *p* < 0.0001; H1299 pERK1/2 (**E**) F(7,16) = 80.83, *p* < 0.0001; and H1299 pAKT (**F**) F(7,16) = 3442, *p* < 0.0001. Significance notation: * adjusted *p* < 0.05 (Dunnett-adjusted; comparisons vs. EGF-only control unless indicated otherwise); unmarked comparisons are not significant.

Pretreatment of A549 and H1299 cells with OXY at all tested concentrations for 3 h did not alter ERK1/2 phosphorylation in response to 5 min of EGF stimulation, and total ERK protein levels remained unchanged (Figure 9A,B,D,E). In contrast, OXY pretreatment significantly suppressed EGF-induced AKT phosphorylation in a dose-dependent manner, without affecting total AKT expression (Figure 9A,C,D,F).

**Figure 10:**
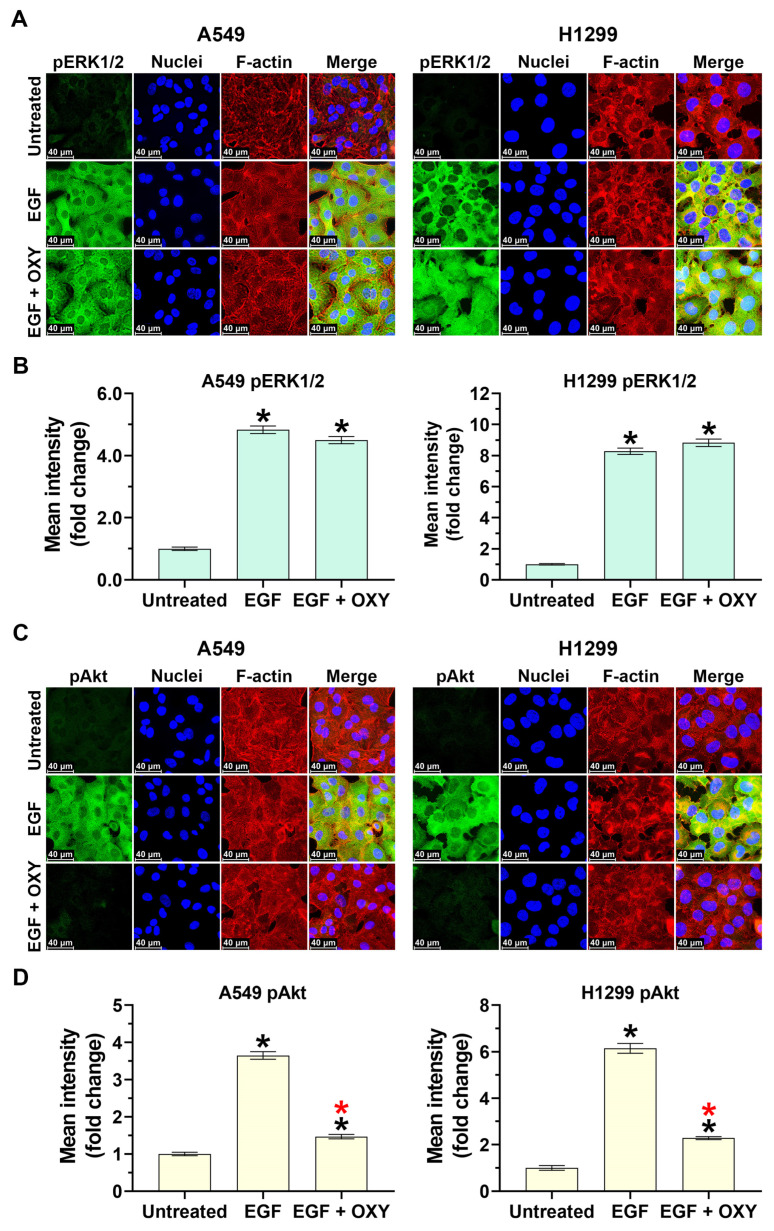
Effect of OXY on EGF-induced ERK and AKT activation in NSCLC cells. (**A**) Representative immunofluorescence images of pERK1/2 (green) in A549 and H1299 cells following EGF stimulation (100 ng/mL, 5 min), with or without OXY pretreatment (200 µM, 3 h). (**B**) Quantification of pERK1/2 fluorescence intensity in A549 and H1299 cells under the indicated conditions (Untreated, EGF, and EGF + OXY). (**C**) Representative immunofluorescence images of pAKT (green) in A549 and H1299 cells under the same treatment conditions. (**D**) Quantification of pAKT fluorescence intensity in A549 and H1299 cells under the indicated conditions. Nuclei were counterstained with DAPI (blue), and F-actin was labeled with phalloidin (red). Scale bar, 40 µm (100× magnification). Statistics (**B**,**D**): Fluorescence intensities were analyzed using ordinary one-way ANOVA followed by Tukey’s multiple comparisons test (family-wise α = 0.05). Significant treatment effects were observed for H1299 pERK1/2 (F(2,156) = 354.7, *p* < 0.0001), A549 pAKT (F(2,313) = 346.2, *p* < 0.0001), and H1299 pAKT (F(2,229) = 389.9, *p* < 0.0001). Significance notation: Black asterisks indicate adjusted *p* < 0.05 vs. Untreated; red asterisks indicate adjusted *p* < 0.05 vs. EGF (Tukey-adjusted). Unmarked comparisons are not significant.

Consistent with these biochemical data, immunofluorescence analysis further supported a selective effect of OXY on AKT signaling (Figure 10). Representative images (Figure 10A,C) showed that EGF stimulation (100 ng/mL, 5 min) increased both pERK1/2 and pAKT signals in A549 and H1299 cells, whereas OXY pretreatment (200 µM, 3 h) did not visibly diminish the EGF-induced pERK1/2 response (Figure 10A). In contrast, OXY reduced the EGF-induced pAKT signal (Figure 10C), which was confirmed by fluorescence quantification (Figure 10D) in both A549 and H1299 cells (Tukey-adjusted). Quantification of pERK1/2 (Figure 10B) supported robust ERK1/2 activation following EGF stimulation and did not indicate suppression of the EGF-induced pERK1/2 response by OXY. Collectively, these findings indicate that OXY selectively interferes with the AKT signaling pathway downstream of EGFR, while leaving ERK1/2 signaling largely intact under these conditions.

## 3. Discussion

Natural polyphenols have attracted considerable attention as potential anticancer agents due to their pleiotropic biological activities and favorable safety profiles [23]. Resveratrol, a well-studied stilbene, has been shown to exert anti-proliferative and pro-apoptotic effects across multiple cancer models, with evidence extending into clinical studies [24,25,26,27]. Oxyresveratrol (OXY), a hydroxylated analog of resveratrol, has been reported to possess anti-oxidant, anti-inflammatory, and anti-tumor properties [14]. However, its mechanistic role in modulating growth factor-driven oncogenic signaling in non-small cell lung cancer (NSCLC) remains poorly defined.

The present study aimed to elucidate the signaling events underlying the anticancer activity of OXY in NSCLC cells, with particular emphasis on its modulatory effects on the epidermal growth factor (EGF)-responsive downstream signaling pathways. Our data showed that OXY significantly decreased metabolic activity and cell counts in both A549 and H1299 cells in a dose-dependent manner. Under EGF-stimulated conditions, dose–response analysis yielded half-maximal inhibitory concentration (IC50) values in the tens of micromolar range in both cell lines when estimated from MTT and from cell count readouts, supporting concordant concentration–response behavior across assays. Notably, statistically significant changes were detectable at lower concentrations (including 12.5–25 μM) for some endpoints, consistent with an exposure-dependent response across the tested range. The reduction in Hoechst nuclear staining and quantitative cell counts corroborated the cytotoxic effect of OXY. Importantly, these effects were observed under conditions of epidermal growth factor stimulation, which normally enhances NSCLC cell survival. This suggests that OXY can counteract EGF-mediated mitogenic signals, thereby impairing tumor cell fitness.

Flow cytometry confirmed that OXY significantly increased apoptotic cell populations in both A549 and H1299 cells. To strengthen apoptosis validation beyond Annexin V/propidium iodide (PI)-based measurements, we additionally quantified active (cleaved) caspase-3 by flow cytometry and assessed poly(ADP-ribose) polymerase 1 (PARP-1) processing by Western blotting. OXY significantly increased active caspase-3 positivity in A549 cells at 50–200 μM and in H1299 cells at 25–200 μM. Consistently, full-length PARP-1 was reduced and cleaved PARP-1 increased (normalized to beta (β)-actin) across overlapping concentration ranges in both cell lines. Together, these findings provide complementary functional and biochemical evidence supporting OXY-induced apoptosis under the experimental conditions tested. Taken together, these data indicate that OXY significantly reduces cellular fitness (metabolic activity and cell number) and promotes apoptosis in both NSCLC cell models under the experimental conditions tested. This finding underlines the ability of OXY to counteract pro-survival signaling cascades, thereby shifting the cellular balance toward programmed cell death. While OXY clearly counteracts pro-survival EGF signaling by promoting apoptosis, it was important to determine whether these effects were mediated through direct inhibition of epidermal growth factor receptor (EGFR) itself. We assessed the impact of OXY on EGFR phosphorylation and receptor dynamics in response to EGF stimulation to better delineate its mode of action. EGFR autophosphorylation is an immediate receptor-proximal event that can occur within seconds of ligand addition; therefore, we examined EGFR phosphorylation in an early seconds-scale window (15–240 s) to capture rapid peak dynamics. In contrast, phosphorylation of downstream effectors such as protein kinase B (AKT) and extracellular signal-regulated kinase 1/2 (ERK) reflects signal propagation and network regulation and is commonly assessed on a minutes-scale window. Accordingly, our phosphorylated AKT (pAKT)/phosphorylated ERK1/2 (pERK1/2) experiments were performed at minutes resolution, which is not inconsistent with the seconds-scale EGFR measurements but instead reflects distinct kinetic windows along the EGFR signaling cascade.

Interestingly, OXY did not interfere with ligand-dependent EGFR phosphorylation at Tyr845, Tyr1045, or Tyr1068, nor did it alter EGFR endocytosis or degradation dynamics. This conclusion is supported by compartment-based quantification of EGFR localization over time. While the mixed-effects model detected modest treatment-related effects in H1299 (perinuclear main effect and a membrane-associated time × treatment interaction), Šídák-adjusted comparisons at individual time points were not significant, indicating no robust OXY-dependent change in EGFR trafficking kinetics under our conditions. These results indicate that OXY does not act as a direct EGFR inhibitor. Although OXY does not directly inhibit EGFR activation or alter receptor dynamics, its ability to counteract pro-survival signaling prompted us to investigate downstream pathways. Given the central role of phosphoinositide 3-kinase (PI3K)/AKT and mitogen-activated protein kinase (MAPK)/ERK cascades in mediating EGF/EGFR-associated signaling outputs relevant to cancer cell survival and proliferation, we next examined whether OXY selectively modulates these signaling nodes to explain its cytotoxic and pro-apoptotic effects.

At the signaling level, OXY selectively suppressed EGF-induced AKT phosphorylation without affecting ERK1/2 activation. This pathway selectivity was supported by both immunoblotting and immunofluorescence analyses. While ERK1/2 activation remained largely preserved after EGF stimulation, OXY consistently reduced EGF-induced AKT phosphorylation; in H1299 cells, a significant overall effect was detected for pERK1/2 imaging quantification, but there was no indication that OXY suppressed the EGF-driven ERK response. Given that AKT is a central regulator of cell survival, metabolism, and cellular fitness programs, its suppression is consistent with the observed reduction in cellular fitness and induction of apoptosis following OXY treatment. The selectivity of OXY for AKT over ERK1/2 suggests that it may act through modulation of PI3K/AKT signaling components rather than broad inhibition of EGFR-mediated pathways. Because we did not perform AKT rescue (e.g., constitutively active myristoylated AKT (myr-AKT)) or AKT-inhibitor phenocopy experiments, the present data support a correlation between reduced AKT phosphorylation and OXY-induced cytotoxic/pro-apoptotic effects rather than a definitive causal dependency. Notably, the pAKT experiments were performed under acute signaling conditions (3 h OXY pretreatment followed by 5 min EGF stimulation), whereas cellular fitness and apoptosis endpoints were assessed after 48 h exposure. Under these acute conditions, OXY significantly reduced EGF-induced AKT phosphorylation at concentrations as low as 25 μM in A549 cells and 12.5 μM in H1299 cells. However, because we did not directly measure viability during the 3 h pretreatment interval, we cannot exclude the possibility that early changes in cell viability or other stress responses contributed to the observed signaling effects. In contrast, 48 h dose–response analyses yielded IC50 values in the ~40–90 μM range across MTT and cell-count readouts. We also note that the pharmacological achievability of the higher in vitro concentrations cannot be established from the present study, as we did not benchmark these exposures against reported plasma or tissue levels of OXY; dedicated pharmacokinetic and in vivo studies will be required to address this point.

Moreover, the selective inhibition of AKT observed in NSCLC cells is consistent with findings across diverse cancer and inflammatory models, where OXY has been shown to interfere with PI3K/AKT signaling [20,21,28,29,30]. This conserved phenotype underscores AKT as a central molecular node affected by OXY, supporting a strong association with its dual anti-cancer and anti-inflammatory activities. By converging on the AKT pathway, OXY treatment is consistently accompanied by a reduction in tumor cell fitness and a dampening of inflammatory responses, thereby reinforcing the robustness and potential biological relevance of its pathway-specific modulation. However, because resveratrol was not tested side-by-side under identical conditions, relative potency or superior bioactivity compared with resveratrol cannot be concluded from the present experiments.

Since the PI3K/AKT pathway represents a central signaling axis implicated in the development and progression of numerous cancers [31,32,33], and given that a wide range of inhibitors targeting this pathway have already been developed [34], our findings position OXY within the category of natural agents that modulate downstream AKT signaling nodes. Taken together, these results support AKT suppression as a conserved phenotypic consequence of OXY treatment, while acknowledging that causality remains to be formally demonstrated. An additional limitation of the current study is that the tested OXY concentration range (12.5–200 μM) includes an upper range (50–200 μM) that is relatively high and may increase the likelihood of non-specific or off-target effects. Notably, the concordance between MTT- and cell count-derived IC50 values provides some reassurance that the concentration–response relationship is not solely attributable to assay-specific metabolic perturbation, although non-specific/off-target activity at higher concentrations remains a concern. We did not include a non-tumorigenic lung epithelial control cell line; therefore, cancer selectivity and therapeutic window cannot be assessed from the current dataset. In addition, we did not benchmark the in vitro exposure range against reported plasma or tissue concentrations of OXY, and thus the in vivo exposure relevance of the tested concentrations remains uncertain. Future studies integrating non-malignant lung models, pharmacokinetic considerations, and direct comparisons with resveratrol will be required to define effective exposure ranges, assess selectivity, and clarify comparative potency.

However, an important limitation of the current study is that a direct, definitive causal relationship between AKT inhibition and the anti-tumor activities of OXY was not established in our models. Fully validating AKT suppression as the mechanistic dependency would require genetic rescue experiments—such as the overexpression of constitutively active AKT (e.g., myr-AKT)—or a comprehensive demonstration that selective pharmacological AKT inhibitors completely phenocopy OXY’s functional effects. Consequently, our data currently establish a strong phenotypic association and correlation rather than an exclusive, defining mechanistic role for AKT inhibition. Future investigations utilizing genetic gain-of-function models are necessary to definitively clarify the exact dependency of OXY on the AKT pathway. Building on these insights, it remains essential to test whether these associations are observed across additional NSCLC molecular contexts.

Collectively, our findings demonstrate that OXY suppresses EGF-induced AKT activation in NSCLC cells, a phenomenon that strongly correlates with reduced cellular fitness (metabolic activity and cell number) and increased apoptosis. Importantly, these effects occurred without interference in EGFR phosphorylation or receptor dynamics, indicating that OXY does not act as a direct EGFR inhibitor but instead selectively modulates downstream signaling. This pathway-specific activity is highly consistent with the cytotoxic and pro-apoptotic phenotypes observed in both A549 and H1299 cells. While our results highlight downstream AKT suppression as a strong association, further studies are required to delineate the precise molecular intermediates involved, perform necessary genetic rescue validations, and evaluate its relevance in animal models of NSCLC, including xenografts or patient-derived systems. These findings provide a rationale for further mechanistic and model-extension studies.

## 4. Materials and Methods

### 4.1. Non-Small Cell Lung Cancer (NSCLC) Cell Culture

A549 and H1299 non-small cell lung cancer cell lines were obtained from the American Type Culture Collection (ATCC) (Manassas, VA, USA). A549 cells were cultured in ATCC-formulated F-12K medium (ATCC 30-2004) (ATCC) supplemented with 10% fetal bovine serum (FBS) (Gibco, New York, NY, USA) and 1% penicillin/streptomycin (Gibco). H1299 cells were cultured in ATCC-formulated Roswell Park Memorial Institute (RPMI)-1640 medium (ATCC 30-2001) (ATCC) supplemented with 10% FBS and 1% penicillin/streptomycin (Gibco). All cells were maintained at 37 °C in a humidified atmosphere containing 5% carbon dioxide (CO_2_). Culture medium was renewed every 2–3 days, and cells were subcultured upon reaching approximately 90% confluency.

### 4.2. Analysis of Metabolic Activity of NSCLC Cells by MTT Assay

A549 and H1299 cells were seeded at a density of 1 × 10^4^ cells per well in 96-well plates and allowed to adhere for 24 h. Cells were then treated with oxyresveratrol (OXY; Merck KGaA, Darmstadt, Germany) at concentrations of 0, 12.5, 25, 50, 100, or 200 µM in serum-free medium (SFM) containing 100 ng/mL epidermal growth factor (EGF; R&D Systems, Minneapolis, MN, USA) for 48 h. MTT (Merck KGaA) solution (5 mg/mL stock in phosphate-buffered saline [PBS]) was added to each well to achieve a final concentration of 0.5 mg/mL, and cells were incubated for 2 h at 37 °C in a humidified atmosphere with 5% CO_2_. After incubation, the MTT solution was discarded, and the resulting formazan crystals were solubilized with 50 µL of 100% dimethyl sulfoxide (DMSO) (Merck KGaA) under agitation at 200 rpm for 5 min. Absorbance was measured at 570 nm using a microplate reader (BioTek Instruments, Winooski, VT, USA).

### 4.3. Cell Apoptosis Assay by Flow Cytometry Analysis

Induction of apoptotic cell death was evaluated by flow cytometry. A549 and H1299 cells were seeded at a density of 1 × 10^5^ cells per well in 24-well plates and allowed to adhere for 24 h. Cells were treated with OXY at the indicated concentrations in the presence of 100 ng/mL EGF for 48 h. Following treatment, cells were harvested by trypsinization, washed twice with PBS, and resuspended in binding buffer (ImmunoTools, Friesoythe, Germany). Apoptotic cells were stained using Annexin V–fluorescein isothiocyanate (FITC) (ImmunoTools) and propidium iodide (PI; Merck KGaA) according to the manufacturer’s instructions. Samples were analyzed using a DxFLEX flow cytometer (Beckman Coulter, Indianapolis, IN, USA), and data were analyzed using CytExpert for the DxFLEX program (version 2.0.0.283). The percentages of apoptotic cells were quantified. Additionally, active caspase-3 was measured using the phycoerythrin (PE) Active Caspase-3 Apoptosis Kit (BD Pharmingen, San Diego, CA, USA) according to the manufacturer’s instructions. Briefly, after treatment with the indicated concentrations of OXY, A549 and H1299 cells were harvested, washed with PBS, and fixed/permeabilized using the kit reagents. Cells were then stained with the PE-conjugated anti-active caspase-3 antibody, protected from light, washed, and resuspended for acquisition. Samples were analyzed by flow cytometry using PE fluorescence. Debris and doublets were excluded by standard gating. Active caspase-3 positivity was determined relative to the control population. Data are reported as the percentage of active caspase-3-positive cells and expressed as percent of the EGF-treated control (OXY 0 µM; set to 100%).

### 4.4. Western Blotting

A549 and H1299 cells were seeded at a density of 1 × 10^5^ cells per well in 24-well plates and allowed to adhere for 24 h. The culture medium was then replaced with SFM for 24 h. Cells were pretreated with OXY at the indicated concentrations for 3 h prior to EGF stimulation. EGF was added at a final concentration of 100 ng/mL to individual wells for the indicated time points. Cells were lysed with lysis buffer, and proteins were subjected to sodium dodecyl sulfate–polyacrylamide gel electrophoresis (SDS–PAGE). Separated proteins were electroblotted onto polyvinylidene difluoride (PVDF) membranes (Merck KGaA) and blocked with 5% bovine serum albumin (BSA; Merck KGaA) for 1 h. Membranes were incubated with specific primary antibodies at 4 °C in a moist chamber overnight. For examining early EGFR phosphorylation dynamics following ligand stimulation, A549 and H1299 cells were seeded in 24-well plates, and EGF was added rapidly to each well to achieve a final concentration of 100 ng/mL. Timing was started immediately upon EGF addition. At the indicated time points (15, 30, 60, 120, and 240 s), medium was aspirated as quickly as possible, and cells were lysed directly in the well by immediate addition of lysis buffer. Lysates were then subjected to SDS–PAGE and electrotransferred onto PVDF membranes. Primary antibodies against total EGFR, phosphorylated EGFR (Tyr845, Tyr1045, Tyr1068), total AKT, phosphorylated AKT (Ser473), total ERK1/2, phosphorylated ERK1/2 (Thr202/Tyr204), and β-actin were purchased from Cell Signaling Technology (Danvers, MA, USA); catalogue numbers and working dilutions were as follows: EGFR (Cat# 4267; 1:1000), phospho-EGFR (Tyr845) (Cat# 6963; 1:1000), phospho-EGFR (Tyr1045) (Cat# 2237; 1:1000), phospho-EGFR (Tyr1068) (Cat# 3777; 1:1000), AKT (Cat# 2920; 1:2000), phospho-AKT (Ser473) (Cat# 4060; 1:2000), ERK1/2 (Cat# 9107; 1:1000), phospho-ERK1/2 (Thr202/Tyr204) (Cat# 4370; 1:2000), PARP-1 (Cat# 9542; 1:1000), and β-actin (Cat# 3700; 1:1000). Membranes were then incubated with secondary antibodies (goat anti-mouse immunoglobulin G (IgG)–IRDye^®^ 800CW and goat anti-rabbit IgG–IRDye^®^ 680RD; LI–COR Biosciences, Lincoln, NE, USA) at room temperature for 1 h; catalogue numbers and working dilutions were as follows: goat anti-mouse IgG IRDye^®^ 800CW (Cat# 926-32210; 1:10,000) and goat anti-rabbit IgG IRDye^®^ 680RD (Cat# 926-68071; 1:10,000). Reactive proteins were detected and quantified using the Odyssey^®^ CLx Imaging System (LI–COR Biosciences). Western blot images were acquired using exposure settings that avoided signal saturation. Only linear, global adjustments were applied equally to the entire image; no background subtraction or selective enhancement was performed. Densitometry was performed using non-saturated images.

### 4.5. Immunofluorescence Study

A549 and H1299 cells were seeded at a density of 1 × 10^5^ cells per well onto glass coverslips placed in 24-well plates and allowed to adhere for 24 h. The culture medium was then replaced with SFM for 24 h. Cells were pretreated with 200 µM OXY for 3 h prior to stimulation with 100 ng/mL EGF for the indicated time points. Cells were fixed with 4% formaldehyde in PBS for 15 min and permeabilized with 0.3% Triton X-100 in PBS for 5 min. Non-specific binding was blocked with 5% BSA in PBS for 1 h. Coverslips were incubated with primary antibodies (Cell Signaling Technology) against total EGFR, phosphorylated ERK1/2 (Thr202/Tyr204), or phosphorylated AKT (Ser473), diluted in 5% BSA/PBS, at 4 °C in a moist chamber for 24 h; catalogue numbers and working dilutions were as follows: EGFR (Cat# 4267; 1:50), phospho-ERK1/2 (Thr202/Tyr204) (Cat# 4370; 1:200), and phospho-AKT (Ser473) (Cat# 4060; 1:400). Samples were then incubated with secondary antibody (Alexa Fluor 488-conjugated anti-rabbit IgG; Merck KGaA, Cat# A11008; 1:400), DAPI (4′,6–diamidino–2–phenylindole; Cell Signaling Technology) for nuclear staining (Cat# 4083; 1 µg/mL), and DyLight–594–Phalloidin (Cell Signaling Technology) for detection of filamentous actin (F-actin) (Cat# 12877; 1:20). Fluorescent signals were detected, and images were acquired using a Leica DMi8 Thunder Imager 3D microscope equipped with LAS X image-processing software (version 3.8.1; Leica Microsystems Ltd., Wetzlar, Germany). Immunofluorescence image analysis and quantification (EGFR, pERK1/2, and pAKT) were performed.

Immunofluorescence images were analyzed using ImageJ (version 1.54t; National Institutes of Health [NIH], Bethesda, MD, USA). For EGFR trafficking, cells were analyzed under three conditions (untreated (UT), EGF, and EGF + OXY) across a 1–60 min time course following EGF stimulation (six indicated time points). For each condition and time point, 20–30 cells were quantified per independent experiment, and results were summarized across *n* = 3 independent biological replicates. Cells were manually outlined to define whole-cell regions of interest (ROIs), and DAPI staining was used to define nuclear ROIs. Perinuclear and membrane-associated regions were defined using fixed-width rings around the nucleus and the cell boundary, respectively; cytoplasm was defined as the whole cell excluding the nucleus/perinuclear region. Background-corrected mean EGFR fluorescence intensity was measured in membrane-associated, cytoplasmic, and perinuclear ROIs and expressed as a fraction of total cellular EGFR for each cell. For visualization, values were expressed relative to the UT baseline (UT set to 1.0) within each compartment, as indicated. For pERK1/2 and pAKT immunofluorescence, background-corrected mean fluorescence intensity was quantified within whole-cell ROIs. Values were expressed as fold change relative to the untreated control (UT set to 1.0). For each condition, 20–30 cells were quantified per independent experiment, and results were summarized across *n* = 3 independent biological replicates.

### 4.6. Statistical Analysis

All quantitative data were analyzed using GraphPad Prism (version 9.0.0; GraphPad Software Inc., San Diego, CA, USA). Data are presented as mean ± SEM from *n* independent biological replicates, with *n*, the statistical test, and the multiple-comparisons procedure specified in the corresponding figure legends. Where analyses relied on assumptions of parametric testing, normality of residuals was assessed in GraphPad Prism (e.g., Shapiro–Wilk test and inspection of Q–Q plots/distribution plots as applicable), and homogeneity of variances was evaluated (e.g., Brown–Forsythe or Bartlett’s test, as appropriate). For experiments with a single independent factor (≥3 groups), data were analyzed by ordinary one-way ANOVA followed by Tukey’s test (all pairwise comparisons) or Dunnett’s test (comparisons to a predefined control), as appropriate (family-wise α = 0.05; multiplicity-adjusted). For experiments with two independent factors, data were analyzed by ordinary two-way ANOVA to test main effects and interactions, followed by multiplicity-adjusted post hoc comparisons (e.g., Dunnett’s comparisons within each level) as indicated (family-wise α = 0.05). For EGFR trafficking time-course analyses (Figure 7), data were analyzed using a mixed-effects model (restricted maximum likelihood; REML) with Geisser–Greenhouse correction, with time and treatment as fixed effects and biological replicate (subject) as a random effect; Šídák-adjusted post hoc comparisons were used for pairwise testing at matched time points (family-wise α = 0.05). A *p*-value < 0.05 was considered statistically significant.

## 5. Conclusions

**Figure 11:**
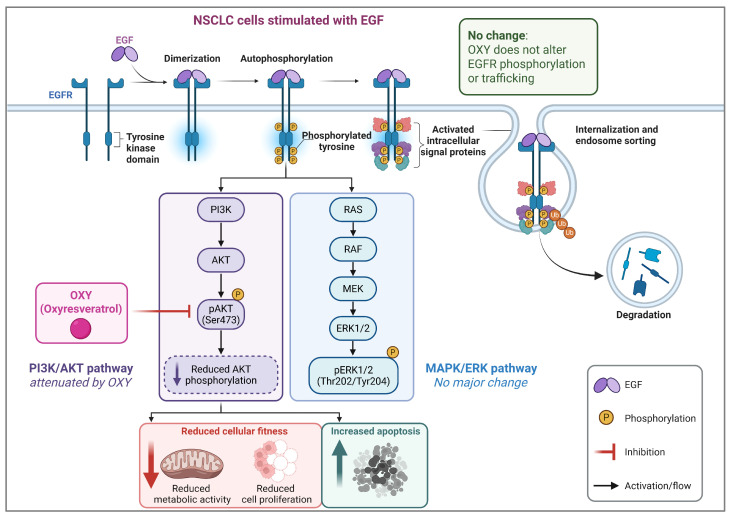
Schematic illustration of the proposed model of OXY action in EGF-stimulated NSCLC cells. Upon EGF stimulation, EGFR activates downstream signaling cascades, including the PI3K/AKT and MAPK/ERK pathways, which promote NSCLC cell survival and proliferation. EGF also triggers rapid EGFR internalization and degradation. OXY treatment does not alter EGFR trafficking and does not affect EGFR phosphorylation/trafficking dynamics or ERK1/2 phosphorylation but selectively suppresses AKT activation, resulting in reduced cellular fitness (metabolic activity/cell number) and increased apoptosis. Created in BioRender. [Nimlamool, W. (2026)] https://BioRender.com/xtlcjn8, accessed on 26 June 2026.

In summary, this study demonstrates that oxyresveratrol (OXY) treatment is associated with reduced cellular fitness and increased apoptosis in non-small cell lung cancer (NSCLC) cells under conditions of epidermal growth factor (EGF) stimulation. At the signaling level, OXY selectively suppressed EGF-induced protein kinase B (AKT) phosphorylation without affecting extracellular signal-regulated kinase 1/2 (ERK1/2) activation or epidermal growth factor receptor (EGFR) dynamics, indicating that its downstream effects are accompanied by pathway modulation rather than direct EGFR inhibition. In addition, OXY did not alter EGFR phosphorylation, further supporting a downstream site of action. The schematic model presented (Figure 11) illustrates these findings, showing how OXY treatment is accompanied by an attenuation of EGF-mediated phosphoinositide 3-kinase (PI3K)/AKT signaling to impair tumor cell fitness while sparing EGFR activation. These findings identify AKT-selective signaling suppression as a reproducible molecular correlate of OXY exposure and provide a rationale for further investigation in more disease-relevant NSCLC models, including xenografts or patient-derived systems. Importantly, the present study does not establish a definitive causal dependency on AKT inhibition; future work employing genetic rescue (e.g., constitutively active AKT) and/or pharmacological phenocopy and/or epistasis experiments using selective AKT inhibitors will be required to validate mechanistic dependency and define upstream molecular targets. Collectively, our results support OXY as a biologically active compound that can counteract EGF-mediated survival signaling outputs, providing a rationale for further preclinical evaluation in NSCLC models. Finally, because these experiments were conducted in EGFR–wild-type NSCLC cellular models, the generalizability of these findings to other molecular subtypes warrants further investigation.

## Data Availability

The original contributions presented in this study are included in the article. Further inquiries can be directed to the corresponding author.

## Abbreviations

The following abbreviations are used in this manuscript:

NSCLC	Non-small cell lung cancer
EGF	Epidermal growth factor
EGFR	Epidermal growth factor receptor Oxyresveratrol
OXY	Oxyresveratrol
MAPK	Mitogen-activated protein kinase
ERK	Extracellular signal-regulated kinases
AKT	Protein kinase B
PI3K	Phosphoinositide 3-kinase
TKI	Tyrosine kinase inhibitor
SFM	Serum-free medium
FBS	Fetal bovine serum
PBS	Phosphate-buffered saline
DMSO	Dimethyl sulfoxide
PI	Propidium iodide
FITC	Fluorescein isothiocyanate
BSA	Bovine serum albumin
WT	Wild type
DAPI	4′,6–diamidino–2–phenylindole
